# Supplementary material for: A capability perspective on sustainable employability: A Dutch focus group study on organizational, work and personal conversion factors
Source: PLoS One. 2022 Oct 21;17(10):e0274000. doi: 10.1371/journal.pone.0274000 (PMC9586339; doi:10.1371/journal.pone.0274000)
Supplement: Attachment — Submitted filename: Response to reviewers 16-7-2022 final.docx [file pone.0274000.s001.docx]

Title: Response to reviewers

Date: 15-7-2022

Dear Editor ,

Thank you for the opportunity to resubmit a revision of our paper. We appreciate the constructive comments provided by the reviewers and have revised our paper accordingly.

The third reviewer has made some important comments about the design of the paper that we aimed to address. We would like to emphasize that in this paper we want to indicate and demonstrate that the capability perspective requires a different kind of scientific analysis; we may not have made that explicit enough. The third reviewer is right that, precisely because of this scientific discussion, we should reflect on the criticism formulated on the capability approach. In the introduction we have now attempted to further formulate and make explicit the value of the capability approach in relation to these critics. In our view, critical reflection on the value of the CA contributes to the scientific discussion on sustainable employability.

We will now detail the changes that we have made to the paper. The authors response will appear in *italic* font. The changes in the paper will appear in ***bold italic font*** with yellow color below. In this rebuttal we refer to the lines of the paper *without the tracked changes.*

**Comments to the Author**

3. Have the authors made all data underlying the findings in their manuscript fully available?

The Plos Data Policy requires authors to make all data underlying the findings described in their manuscript fully available without restriction, with rare exception (please refer to the Data Availability Statement in the manuscript PDF file). The data should be provided as part of the manuscript or its supporting information, or deposited to a public repository. For example, in addition to summary statistics, the data points behind means, medians and variance measures should be available. If there are restrictions on publicly sharing data—e.g. participant privacy or use of data from a third party—those must be specified.

Reviewer #1: Yes

Reviewer #2: Yes

Reviewer #3: No

Authors response: *We have stored anonymized raw transcripts of the focus groups in the data repository of Tilburg University. On reasonable request, we provide open access to scholars interested in the data.*

5. Review Comments to the Author

Please use the space provided to explain your answers to the questions above. You may also include additional comments for the author, including concerns about dual publication, research ethics, or publication ethics. (Please upload your review as an attachment if it exceeds 20,000 characters)

**Reviewer #1:**

1. Introduction: it is of interest to note that Jahoda did her seminal studies in the 1950s and 1960s, hence, the statement that nowadays other values are important needs more careful consideration!

*Response Authors: Thank you for your comment. We have adapted this notion with a more careful formulation. (line 52-54)*

“Jahoda was one of the first who conducted research on work values and highlighted income as the single most important work value.^4^ Albeit, she considered other relevant values as ‘latent values’. Nowadays, we acknowledge that indeed many other values such as security, recognition and meaning play an important role.”

2. Introduction: I am not sure that JDR model has shifted from outcomes of interventions...I would argue that the JDR model is used most in observational studies rather than intervention evaluation.

*Response authors: Thank you for this important remark. As we have changed the framing of the article to concretize our research goal, the sentence you refer to has been removed from the introduction.*

3. Introduction: the central aim is interesting, but comes a bit out of the bleu. What is already known? I would invite the authors to expand a bit on the novelty of the current study.

*As a response to your feedback, we have aimed to better frame the goal of our study, including highlighting the novelty of the study. See introduction on “sustainable employability”, line 48-120*

4. Methods: i feel that the authors use the term outcome very loosely...eg line 173 (see also remark 2)

*Authors response: Thank you for your remark, we agree that “outcome” might not be the right term. We have aimed to use a different term such as results, or we have removed the term outcome in the full manuscript.*

5. Methods: how many participants were included in total, were focus groups done per company (as some of these choices were done as part of the design of the study, please shortly mention this in the method section)?

6. Results: participants, any idea about the educational background?

*Response authors on point 5 and 6.: We have included additional information on the descriptive statistics of the participants. See table 1 in the method section, line 202*

7. Results: I suggest to include a summarizing table of the conversion factors at three levels.

*Authors response: Thank you for this advice. We have included a summarizing table of conversion factors in the result section, see table 2, line 248*

8. Results: Sometimes the language is a bit confusing, eg line 327 you start with limitations, and then you continue with a positive remark. Thus, the word limit should really be influenced..eg line 346 you introduce the phrase 'to get the grid around', which is difficult to interpret.

*Authors response: Thank you for your feedback on the use of confusing language. We have removed the sentence on limitations and changed this specific part to more formal language (line 317).*

“Due to ongoing challenges to complete work schedules, there was simply no time for additional courses, unless in private time. “

9. Results: the social contacts should be explained more for their target (colleagues, supervisor, clients etc). Contacts with the the latter group can be, according to literature, both be positive and negative.

*Authors response: We have made an effort to specify social contacts for their “target”, as these can indeed be negative or positive. See line 347*

“In the insurance group, employees discussed their preferred choice to work as a team. The good social contacts *with colleagues and clients* in this team facilitated many of the work values discussed in this group on a daily basis.”

10. Results: line 396 is strange...you start with defining personal level, and then present between group (which by definition is not about individuals)

*Authors response: We agree that by definition, personal conversion factors belong to the individual participant. We have changed the text, line 365*

“On the personal level, workers seemed to differ in how they contributed to the realization of their values in work. “

11. Results: definition problem: is lack of challenge in the job an individual attribute?

*Authors response: In terms of the capability approach, this is dependent on the specific context, see additive elaboration in the introduction, line 93-101*

12. Discussion: I think that the discussion should be more balanced with respect to evaluation of the direction of the conversion factors, e.g. hierarchy is presented as a problem, and this will depend most likely on educational/professional level of the participants, and also on cultural context. Thus, I would suggest to identify factors rather than interpreting their direction.

*Authors response:* Thank you for your feedback. We have aimed to find more balance by including more types of possible biases in focus groups and by adding the following remark to our discussion, lines 505

“Also educational background and professional level of participants might relate to differences in reflecting on conversion factors by the group.”

13. Discussion: the section on agency starts with a theoretical explanation, which should either follow from the results, or be introduced in the methods.

*Authors response: Thank you. We agree, though after thoughtful consideration, we have decided to remove the part on agency from the paper.*

14. The results are about social contacts, in the discussion this seems ot be interprted as social coherence, which is something else.

*Authors response: Thank you. We agree social contacts is something different as social coherence. We have taken the term social coherence out of this paper, and changed it into social contacts throughout the paper.*

**Reviewer #2:**

The reviewed paper presents an important and up-to-date issue of a capability perspective on sustainable employability. The discussed topic requires further scientific exploration.
The article brings sufficient contribution to the development of knowledge in the concerned area. The article closely corresponds to the topic specified in the title. In this article all the issues were discussed in an understandable manner.
The literature selection presents current articles, but some of the root articles are also implemented.

I suggest supplementing the subject literature based on the latest publications from this area. The purpose of the article should be clearly defined. The argumentation and contribution of the paper is on a good level. The conclusions reflect the research results and are linked to the rest of the paper.

*Authors response: Thank you for your valuable feedback and comments. We have added the latest publications in the literature on the conceptualization of sustainable employability to clarify the purpose of the article* in the introduction (line 48-179)

**Reviewer #3**  
Thank you for the opportunity to review this interesting qualitative paper on the capability approach and sustainable employability. The paper deals with a generally important topic and offers some insights into elements that relate to it. In that sense, the paper is an interesting read. However, in my opinion, the current framing of the paper does not do a convincing job at testing a theory or contributing to theory building (even if it does suggest to do so). The evidence presented and the way it is connected to conclusions, existing evidence, and the theory it is supposed to contribute to are insufficiently convincing. I think this could be solved by implementing a better framing and connecting to the existing sustainable employability literature better. However, it is at the same time unclear to me whether the quality of the data is sufficient to make claims to allow for positioning the identified factors as conversion factors. In sum, although the paper is interesting, I do think the authors have work to do regarding its framing and clarity of the methodological approach and connecting finding to results and conclusions that the paper sets out to offer. Below I offer several comments that are intended to aid the authors in doing so. I hope these are helpful.

*Authors response: Thank you for your feedback. You have made important comments about the design of the paper that we have addressed. In addition, we would like to emphasize that in this paper we want to indicate and demonstrate that the capability perspective requires a different kind of scientific analysis; we may not have made that explicit enough. You are right that, precisely because of this scientific discussion, we should reflect on the criticism formulated on the capability approach in the field of SE. In the introduction we have now attempted to further formulate and make explicit the value of the capability approach in relation to these critics.*

Introduction / theory
A major issue with the current paper is that it is focused on sustainable employability but only incorporates a very limited account of that literature. There have been marked developments after the emergence of the capability approach (see for an elaborate critical review specifically: [https://doi.org/10.3390/su12166366](https://urldefense.com/v3/__https:/doi.org/10.3390/su12166366__;!!P3D15CNZqp7UcEw!ePLLTR7QY3NoO5O2jenv0qhO3V0qwfy_XiktJY7NXuTP8F1xA9aV3MbTEp8wvwW-sSdMPELBBSLxfaGwczUaykk$)). The capability approach has been heavily criticized in the literature and several other approaches (that are somewhat compatible with the capability approach) have emerged, but the present paper does not relate to that discussion in any way. Certainly there are ways out of that with the capability approach still having relevance in the context of sustainable employability, but to have a convincing paper that deals with the issue of sustainable employability I think the authors need to connect to the state of the art thinking on this concept.

*Authors response: Thank you very much for the elaborate and valuable feedback on our paper. We agree that the paper might profit extensively from a better framing, which we have aimed to achieve by adding a paragraph on differences in conceptualizing and operationalizing of sustainable employability in the introduction. As this paper builds on the conceptualization and operationalization of sustainable employability in terms of capabilities, our goal is to build theory on the nature of conversion factors in work in this respect. See introduction, (lines 48-179)*

The paper incorporates several theories and concepts from the fields of work and organizational to connect to sustainable employability. However, the paper does not do a thorough job at explaining these concepts and theories and connections are very loosely made (e.g., ‘positive psychology’ which is a somewhat hollow and broad term; ‘self-determination theory’ which is not explained, ‘house of workability’ which offers little structure in terms of modelling relationships and is also not very clearly explained, the ‘job demands resources model’ which is not explained, ‘positive employability outcomes’ is used but then covers things not traditionally associated as such, ‘person-job fit’ is highlighted but not properly explained). Consequently, the point of incorporating these theories / concepts is not clear to me and I am not convinced that readers who are not very familiar with these theories and concepts can easily follow the line of reasoning. Related to the previous point, if a better connection would be made to the sustainable employability literature, this haphazard discussion of several (already debated) concepts would not be necessary and could be dealt with more efficiently. On a somewhat also related note, the introduction paragraph where this takes place is rather lengthy.

*Author’s response:* *Thank you for your response. Because we have framed the introduction differently, we have developed a line of reasoning in which the concepts and models referred to in our previous version of the paper have been largely removed or placed in our current framing. We have referred to the JDR and Ilmarinen to illustrate our argument that value plays an increasingly important role also in these in the field of work and health important models.*

The discussion on the capability approach is easy to follow, but seems a bit imprecise and diverging from original formulations in key papers proposing that approach to sustainable employability. I recommend a more precise discussion and handling of key concepts. There is also some arguable discrepancy between how resources are framed within the capability model and how capabilities are actually measured in the original capability set. Statements suggesting that the capability approach does not focus on resources are therefore rather debatable.

*Authors response:* *Thank you for your feedback. We have worked on the handling of capability elements throughout the paper, especially in the new theoretical background section, to synchronize with the key paper of van der Klink et al. (2016). Lines 123-179*

It is somewhat strange that the paper has a paragraph on ‘operationalizing the capability set’ because this has been done in a previous paper already that this paper draws on. With this framing of the paragraph, it is not clear to me as reader what I need to do with the ‘operationalizing’ idea in this context. Perhaps a different frame would help.

*Authors response: Thank you, we agree. We have changed the heading. See line 143*

*“The capability approach applied to the field of SE”*

The paper states ‘Although the capabilities of Dutch employees are established’. I find that a rather bold and highly debatable statement. What does it even mean? How can such a claim be made on the basis of a single reference? In this same paragraph there is marked repetition of previously made points on conversion factors. The ‘therefore’ sentence after this paragraph is also oddly disconnected from the paragraph itself, and arguably its content does not follow from the preceding paragraph at all.

*Authors response: Thank you, we agree this claim is debatable and the argument has not been formulated properly. We have removed the sentence and reformulated the paragraph on conversion factors. Line 167-179*

*Conversion factors*

The relationship between personal and contextual factors which convert resources into capabilities has not deserved much attention in the capability- and the work and health literature. Following Robeyns^20^ and van der Klink et al.^2^, it is assumed that in work, three levels of conversion are of importance. Organizational conversion factors relate to policies of the company concerning the work of the employee and include societal factors (such as legislation). Work conversion factors are related to the direct work surroundings of the worker which impact performing of daily work tasks. Personal conversion factors are related to the background of the participant applicable to the work context, such as social skills, capacity to perform tasks, activities and take certain roles, sociocultural values motivation and energy level. In work, for example, if one has the value of developing knowledge and skills, the way a company facilitates courses is an organizational conversion factor, the manager that allows time and financial resources to join the course are work conversion factors and the motivation to study and prepare the courses in private time are personal conversion factors. Without these conversion factors it is not possible to convert resources into capabilities.

This in itself would not be a big issue, but what is currently still missing in the paper is a clear motivation as to why we should care about this topic at all (what is the issue at hand, why is it so relevant to know about capabilities?) and what important research gap is covered (what do we really know about the capabilities, why don’t we know the specific thing this study investigates, and why is that important to know?). Consequently, the contribution of the study also remains rather vague; what do researchers in this area or society in general stand to gain from this paper? I think these elements should be articulated much more explicitly and convincingly than is currently the case.

*Authors response: Thank you, your critical remarks on the motivation on the paper has challenged us to formulate more precisely why we are convinced of the scientific value of this paper. See the introduction lines 48-120. Furthermore, we refer to the discussion, more specifically lines 443 - 457*

*Normativity, contextuality and diversity*

In the introduction we have introduced the CA based on normativity, contextuality and diversity. In this focus group study the main goal was to identify conversion factors in relation to work value realization. In terms of normativity, this focus group method seems also suitable to be used as a first step for an intervention study or action research to increase SE within a company. In relation to contextuality, this study seems to ascribe that conversion factors at multiple levels influence value achievement of the worker. As an example, it was seen that for each focus group limiting conversion factors could be on all three levels of conversion. In one group the organization could limit the value to make one’s own decisions and plans, in another group, at the work level, the communication between colleagues could facilitate the application of knowledge and skills and on the personal level some employees seemed better in others to find informal ways to achieve values such as setting one’s own goals. In terms of diversity, the findings suggest that specific characteristics seem important to realize values. This supports the need of tailored made analyses and interventions to support the diversity of workers working in the same work context. Instead of facilitating or limiting factors found on a group level which might be determinants of SE in a positivistic sense, conversion factors have been found which seemed essential in the process of value realization of employees in their specific situation.

Several conversion factors are mentioned, but there is no clear argumentation as to why these factors would function as such. An example would be ‘age’, why would that be such an important conversion factor? And how does that work? I think the paper would benefit from an example or a more convincing explanation of this to help the reader understand conversion factors better.

*Authors response:* *Thank you for pointing to the lack of clarity on the concept of conversion factors. We have added additional information on the conceptualization of conversion factors in the introductory section, as well as in the paragraph on conversion factors. In addition, we added an example, to illustrate the levels of conversion. Line 175-179. In the discussion, elaboration on normativity, contextuality and diversity the concept of conversion factors is also discussed 443 -457.*

In work, for example, if one has the value of developing knowledge and skills, the way a company facilitates courses is an organizational conversion factor, the manager that allows time and financial resources to join the course are work conversion factors and the motivation to study and prepare the courses in private time are personal conversion factors. Without these conversion factors it is not possible to convert resources into capabilities.

*Normativity, contextuality and diversity*

In the introduction we have introduced the CA based on normativity, contextuality and diversity. In this focus group study the main goal was to identify conversion factors in relation to work value realization. In terms of normativity, this focus group method seems also suitable to be used as a first step for an intervention study or action research to increase SE within a company. In relation to contextuality, this study seems to ascribe that conversion factors at multiple levels influence value achievement of the worker. As an example, it was seen that for each focus group limiting conversion factors could be on all three levels of conversion. In one group the organization could limit the value to make one’s own decisions and plans, in another group, at the work level, the communication between colleagues could facilitate the application of knowledge and skills and on the personal level some employees seemed better in others to find informal ways to achieve values such as setting one’s own goals. In terms of diversity, the findings suggest that specific characteristics seem important to realize values. This supports the need of tailored made analyses and interventions to support the diversity of workers working in the same work context. Instead of facilitating or limiting factors found on a group level which might be determinants of SE in a positivistic sense, conversion factors have been found which seemed essential in the process of value realization of employees in their specific situation.

The paper selects people from different sectors, but the logic behind selecting those sectors is never presented. Why these, why not others?

The build up of the sample is completely unclear after reading the methods section. Information on the sample is needed there. How purposive was the constellation of each focus group? Was there a specific reason for a certain build-up, was this build-up adequate for having the discussion?

Social desirability, presentation bias, and rationalizations potentially strongly limit the validity of statements made by participants and consequently conclusion drawn from them. More so than aspects mentioned.

*Authors response:* *We have adapted the sampling procedure in the method section to more fully elaborate on our sampling strategy. See line 190-200. In addition we have broadened our discussion of potential bias on our findings.*

*Participants*

Focus group participants were recruited in different occupational sectors in order to include a broad range of the overall Dutch labour market. In this way, we aimed to include different work contexts, including organizational conversion factors. Direct contact persons of the researchers at seven Dutch companies in different occupational sectors were approached by phone. These contact persons were asked to sample a group of collaborating colleagues within the company, as it was assumed these colleagues would be better able than non-collaborating colleagues to discuss conversion factors at the work floor. Furthermore, individuals were eligible to participate when speaking Dutch fluently, were aged between 18 and 67 and interested to participate in discussion on the topic of sustainable employability in their company. The groups needed to include a minimum of five participants. See table 1 for the general characteristics of the focus groups.

*In the discussion we have added a remark on the adequateness of this build up for having discussions and added other forms of bias, which could have impacted the findings. (lines 492-506)*

As the groups differed in the number of participants, this might have influenced the involvement of participants in the larger groups.^34^ In the literature six to ten people per group has been found appropriate for discussion.^35^ In the larger groups such as the HRM group, it was observed there was less dynamic discussion on personal conversion factors, whereas in smaller groups with workers that were more familiar to each other, such as the elderly care group, personal conversion factors were discussed more openly.

It is known that in focus groups different biases are faced, which might have impacted on the findings. ^36^ Participants tend to take many assumptions of others for granted much more easily and express culturally appropriate views only (acquaintance bias). In addition, there might have been a social desirability bias, towards colleagues and interviewers, as the meeting has been framed as a discussion on sustainable employability. Another possible factor impacting the findings in certain focus groups has been dominance bias (by which dominant individuals shape the conversations) and the halo bias (perceived status of participants influences the openness of sharing thoughts). Despite the Dutch horizontal consensus economy, employees higher in the hierarchy within the company might have naturally influenced the opinions and reflections of workers lower in the hierarchy during the focus group. Also, educational background and professional level of participants might have affected the depth of reflection on essential conversion factors by the group.

In the methods section, the paper states ‘We chose focus groups as a methodology to identify conversion factors, because it seems reasonable to take a similar strategy in line with methodologies in the capability literature as stated above.’ This is an odd line of argumentation; one should choose the method / design because it is best suited to answer the research question, not because ‘others did this too’.

*Authors response:* *Thank you for pointing us to this illogical line of reasoning. We have changed the line of reasoning accordingly. Lines 183-189*

We chose focus groups as a methodology to identify conversion factors, because focus groups have the advantage of discussion between a diverse range of workers with different tasks and roles within a company. Furthermore, focus groups provide a room for dialogue, not only to identify capabilities, but also for reflection on achieving values of different people and what possibly facilitates and limits them in achieving values. Moreover, the focus groups might provide immediate suggestions for interventions for the group or individual workers. The consolidated criteria for reporting qualitative research (COREQ) were used to comprehensively report the focus group process.^27^

Is ‘Focus group interviews’ an accurate and commonly used term? I am not sure.

*Authors response: We have changed “focus group discussions” and “focus group interviews” into ‘focus groups’ throughout the paper.*

Is there a coding tree? Is there a protocol for the focus groups available to get insight into questions / discussion topics used?

*Authors’ response: We have used a Dutch topic list for the focus group interviews. When deemed necessary by reviewers and editor, we will translate these and add them as an appendix.*

In the Results section, it is odd that the research question is stated again, unnecessary repetition.

*Authors response: Thank you. We have removed the research question in the result section.*

The results section connects some interpretations to industries/sectors; how adequate is the sampling approach for doing that? It doesn’t seem to me like the sample is built up in such a way that such conclusions are possible.

*Authors response: We fully agree there should not be any impression that findings are generalized to an entire organizational sector. We have aimed to acknowledge your feedback by adding a informational sentence in the beginning of the results section. See line 244.*

“For practical reasons, focus groups will be either consistently referred to as the common function of the participants or the organizational sector.”

How convinced are the researchers that the ‘conversion factors’ listed are really ‘conversion factors’ because to me they are known aspects that make a work situation nice or horrible. What evidence do we have for the framing of conversion factors? The quotes used don’t seem to explicitly go into that direction (i.e., taking the idea that conversion factors represent the situation of a resource being available to an extent and then the things listed as conversion factors determine the extent to which that resource can be used to realize a certain value).

*Authors response:* *Thank you for your feedback, which is in line with earlier feedback on the nature of conversion factors. We have aimed to better frame this nature in the introduction section (lines 167-179), and it is also discussed in the discussion (lines 443-457).*

What is the ‘work level’? It seems more like ‘group’ / ‘team’ level factors discussed there .When I think of the work level, I would think of the nature of work and tasks that individuals perform (either alone or in teams).

*Authors response: In response to your feedback we have clarified the three levels in the introductory section, lines 170-175.*

Organizational conversion factors relate to policies of the company concerning the work of the employee and include societal factors (such as legislation). Work conversion factors are related to the direct work surroundings of the worker which impact performing of daily work tasks. Personal conversion factors are related to the background of the participant applicable to the work context, such as social skills, capacity to perform tasks, activities and take certain roles, sociocultural values motivation and energy level.

The discussion section talks about agency, but where in the data do we see evidence of ‘agency’. Current write up is not convincing here because the connection is insufficiently explicit. And relatedly, how is this different from the vast knowledge on autonomy?

*Authors response: Thank you, we agree and have removed the paragraph on agency from the paper.*

What is social coherence? It seems like a complex concept but it is not clearly defined where it is discussed. And then, what is the conclusion here based on specifically.

*Authors response: Thank you, we agree this concept is too complex to use in this paper. We have removed social coherence and changed it to social contacts throughout the paper.*

The JDR paragraph is somewhat too limited. It offers an important (and previously formulated) critique to the JDR (i.e., demands and resources are non-concepts, because whether something is a demand / resource depends on how a person sees it). It could make this point more clear and connect to previous literature on this idea.

*Authors response: Thank you. We have decreased the connection between JDR & CA in this paper as it does not serve the current framing. In a way we refer to the JDR model in de paragraph in the discussion on the concept of value in relation to SE (lines 473-480).*

*The concept of value in relation to SE*

In the introduction it was stated that values are increasingly important in the field of work and health and in SE. The results from this study offer preliminary support that values indeed deserve more attention in the conceptualization of SE. If high work demands fully align with the work values of the employee, they might become facilitative work conversion factors which increase SE, such as perceived by certain workers in the higher education group. On the other hand, when a large share of demands does not contribute to value realization, this seems to negatively impact the achievement of values such as in the facility management group.

‘valid predictors of sustainable employability’. Read the suggested literature on sustainable employability to see that such a statement cannot be convincingly made at all, unless there is a profound quantitative study that longitudinally connects aspects to development in sustainable employability indicators over time.

*Authors response: we agree this formulation is debatable and does not fit in the line our paper. We have removed the sentence.*

Abstract is very clear and well-written, but it would be informative to know a bit more about the data source (e.g., how many people, how long the discussions, which sectors represented).

*Authors response: We have adapted our abstract accordingly. See lines 15-47*

**Abstract**

**Objective**

In the field of work, there is a shift towards more value-based approaches to study the sustainable employability of the present-day worker. The capability approach offers a value based and innovative conceptualisation and framework of sustainable employability characterized by contextuality, normativity and diversity. The capabilities of Dutch employees have been established and validated, yet it is not known which conversion factors on a personal, work and organizational level enable employees to achieve value in work in different Dutch occupational sectors.

**Methods**

Our qualitative approach included seven focus groups in different occupational sectors including elderly care, higher education, insurance work, facility management and the oil-, car- and chemical industry. Each focus group included 5-11 participants and took approximately one and a half hour. A qualitative content analysis was used to analyse the data, by combining deductive and inductive coding respectively. Deductive coding involved assigning themes to the conversion of resources into capabilities at the organizational, work and personal level.

**Results**

On the organizational conversion level, important themes were cultural aspects, power relations, shortage of personnel and policies for self-management. On the work conversion level, social contacts, communication and workload, tasks and schedules were identified. Social contacts were described as a work value in itself, but also conditional for achieving other work values. On the personal conversion level, experienced work stress, motivation and the ability to achieve values informally within the company.

**Conclusion**

From our findings it follows that focus groups are sensitive to identify conversion factors on all three levels of conversion. In addition, companies and their employees might effectively increase work capabilities by being sensitive to all three conversion levels simultaneously. Further research is necessary to study the effect of a capability-based intervention at the work floor.

It is too me very unclear what this study adds to existing literature. Even after reading the conclusions I have the impression that we learned nothing new. Maybe this is false, but it too a large extent relates to the fact that things are not adequately framed and connected to evidence to allow for specific novel conclusions and insights regarding conversion factors within the capability approach.

*Authors response: Thank you for your extensive remarks to improve this paper. We hope our adaptations have helped to display the additive value of the paper.*
